# Broad Impact of Exchange Protein Directly Activated by cAMP 2 (EPAC2) on Respiratory Viral Infections

**DOI:** 10.3390/v13061179

**Published:** 2021-06-21

**Authors:** Eun-Jin Choi, Wenzhe Wu, Xiaoyan Cong, Ke Zhang, Jiaqi Luo, Sha Ye, Pingyuan Wang, Adarsh Suresh, Uneeb Mohammad Ullah, Jia Zhou, Xiaoyong Bao

**Affiliations:** 1 Department of Pediatrics, The University of Texas Medical Branch, Galveston, TX 77555-0372, USA; euchoi@utmb.edu (E.-J.C.); wenwu@utmb.edu (W.W.); xycong@163.com (X.C.); kezhang@utmb.edu (K.Z.); luojq8@mail2.sysu.edu.cn (J.L.); shye@utmb.edu (S.Y.); as210@rice.edu (A.S.); uu12@utexas.edu (U.M.U.); 2 Department of Pharmacology & Toxicology, The University of Texas Medical Branch, Galveston, TX 77555-0372, USA; piwang@utmb.edu (P.W.); jizhou@utmb.edu (J.Z.); 3 Department of Kinesiology, Rice University, Houston, TX 77005, USA; 4 College of Natural Sciences, The University of Texas at Austin, Austin, TX 78712, USA; 5 Sealy Center for Molecular Medicine, The University of Texas Medical Branch, Galveston, TX 77555, USA; 6 The Institute of Translational Sciences, The University of Texas Medical Branch, Galveston, TX 77555, USA; 7 The Institute for Human Infections and Immunity, The University of Texas Medical Branch, Galveston, TX 77555, USA

**Keywords:** adenovirus, human metapneumovirus, respiratory syncytial virus, EPAC2, viral replication, host cellular response

## Abstract

The recently discovered exchange protein directly activated by cAMP (EPAC), compared with protein kinase A (PKA), is a fairly new family of cAMP effectors. Soon after the discovery, EPAC has shown its significance in many diseases including its emerging role in infectious diseases. In a recent study, we demonstrated that EPAC, but not PKA, is a promising therapeutic target to regulate respiratory syncytial virus (RSV) replication and its associated inflammation. In mammals, there are two isoforms of EPAC—EPAC1 and EPAC2. Unlike other viruses, including Middle East respiratory syndrome coronavirus (MERS-CoV) and Ebola virus, which use EPAC1 to regulate viral replication, RSV uses EPAC2 to control its replication and associated cytokine/chemokine responses. To determine whether EPAC2 protein has a broad impact on other respiratory viral infections, we used an EPAC2-specific inhibitor, MAY0132, to examine the functions of EPAC2 in human metapneumovirus (HMPV) and adenovirus (AdV) infections. HMPV is a negative-sense single-stranded RNA virus belonging to the family Pneumoviridae, which also includes RSV, while AdV is a double-stranded DNA virus. Treatment with an EPAC1-specific inhibitor was also included to investigate the impact of EPAC1 on these two viruses. We found that the replication of HMPV, AdV, and RSV and the viral-induced immune mediators are significantly impaired by MAY0132, while an EPAC1-specific inhibitor, CE3F4, does not impact or slightly impacts, demonstrating that EPAC2 could serve as a novel common therapeutic target to control these viruses, all of which do not have effective treatment and prevention strategies.

## 1. Introduction

Acute lower respiratory tract infections (RTIs) have been one of the top five causes of death globally in adults and children, which is estimated to lead to nearly 4 million deaths annually [1,2]. Young children, the elderly, and immunocompromised patients are particularly susceptible to severe RTIs. It has been reported that two-thirds to three-fourths of cases of acute respiratory illness are caused by viruses [3]. More than other factors, viral-induced respiratory diseases have a significant impact on public health due to their rapid spread across the globe. For example, since the emergence of severe acute respiratory syndrome coronavirus 2 (SARS-CoV-2) in December 2019 [4,5], there were about 91 million confirmed infections and 2 million deaths worldwide as of 14 January 2021 [6]. The COVID-19 pandemic has been generating health, economic, and social crises worldwide [7]. Given that multiple viruses are the causative agents of human respiratory infection and co-infection has been continuously reported, the development of a novel treatment modality that has a broad-spectrum antiviral effect is urgently needed.

Human metapneumovirus (HMPV), which belongs to the Pneumoviridae family, is a negative-sense single-stranded RNA virus. Similar to respiratory syncytial virus (RSV), it is also the leading cause of lower RTI in young children, high-risk adults, and the elderly [8,9,10]. In some regions, HMPV was reported to be only secondary to RSV infection [11]. Overall, these viruses are responsible for a significant health burden of disease in children under five years of age in terms of hospitalizations, emergency/urgent care visits, and clinic visits [12,13]. Co-infection of HMPV and RSV has been demonstrated to be associated with a higher risk of ICU admission in children under five years [14] and increased disease severity in infants [15]. In addition to these RNA viruses, double-stranded DNA adenovirus (AdV) is also a common cause of RTI in children and adults [16,17]. Adenovirus infection can cause significant morbidity and mortality in young children and immunocompromised patients [18,19,20]. Unfortunately, there are no effective antivirals or vaccines available for the prevention or treatment of these viral infections. Palivizumab, a monoclonal antibody (mAb) targeting fusion protein of RSV, was licensed in 1998 for prophylactic use in high-risk infants. However, it is not very cost effective with a limited application to high-risk infants in their first period of exposure to the RSV season [21].

Exchange protein directly activated by cyclic AMP (cAMP) (EPAC) is a downstream effector of cAMP, consisting of two isoforms, EPAC1 and EPAC2, in mammals [22,23]. Compared with protein kinase A (PKA), EPAC is a relatively new effector family. Cumulative findings have shown the important function of EPAC in various diseases including heart failure, cancer, neurological disorders, diabetes, inflammation, and bacterial and viral infections [24,25,26,27,28,29]. For this reason, several activators and inhibitors, targeting EPAC1, EPAC2, or both, have been continuously developed [30,31,32]. We have recently investigated the effect of EPAC on RSV infection and found that EPAC2 regulates RSV replication and its associated innate immune response [33]. Earlier than our findings, another group demonstrated the impact of EPAC1 on Middle East respiratory syndrome coronavirus (MERS-CoV) infection [28], with more recent publications demonstrating the impact of EPAC1 on other viral infections [34,35]. However, the significance of EPAC2 in viral infections is still limitedly reported. Overall, research on the function and significance of EPAC in virus infection is recently being explored and remains to be investigated in many other viral infections.

Herein, we aim to examine the broad-spectrum effect of EPAC on respiratory virus infection. Since both HMPV and AdV are major causes of RTI in humans, we selected HMPV and AdV as a representative RNA and DNA viruses, respectively, for the study. Using EPAC1- and EPAC2-specific inhibitors [36,37], we found that the EPAC2-specific inhibitor MAY0132 significantly suppressed HMPV and AdV replication and cytokine/chemokine induction in both immortalized and primary epithelial cells, while the EPAC1-specific inhibitor CE3F4 played a minimal role. Furthermore, EPAC2 inhibitors were highly effective in interfering with the polyinosinic–polycytidylic acid (poly I:C)- or TNF-α-induced NF-κB activation. Taken together, our results propose the potential of EPAC2 as a novel therapeutic target against multiple respiratory viral infections to control not only virus replication but also cytokine/chemokine induction.

## 2. Materials and Methods

### 2.1. Cells, Virus, and Reagents

A549 (human alveolar type II-like epithelial), HEp-2 (human epithelial type 2), LLC-MK2, and 293 (human embryonic kidney epithelial) cells were obtained from the ATCC (Manassas, VA, USA) and maintained as previously described [33]. Primary cultured human SAE (small airway epithelial) cells were purchased from Lonza (distributed via Fisher Scientific, Pittsburgh, PA, USA). The isolate HMPV CAN-83 was propagated in LLC-MK2 cells at 35 °C in the absence of serum and the presence of 1 μg/mL of trypsin and was sucrose-purified, as previously described [38,39]. HMPV titer was determined by immunostaining in LLC-MK2 cells using polyclonal rabbit anti-HMPV antibody and streptavidin-peroxidase-conjugated secondary antibody (Sigma-Aldrich, St. Louis, MO, USA), as previously described [40]. Human AdV serotype 5 was kindly provided by Dr. Matthew D. Weitzman from the Children’s Hospital of Philadelphia. AdV yield was determined by plaque assay using crystal violet staining as previously described [41]. RSV long strain was propagated in HEp-2 cells at 37 °C and purified by sucrose gradient as described previously [33,42]. RSV titer was determined by immunostaining in HEp-2 cells using polyclonal biotin-conjugated goat anti-RSV antibody (Bio-Rad, Hercules, CA, USA) and streptavidin-peroxidase-conjugated secondary antibody, as previously described [33,42]. CE3F4 and H89 were purchased from Tocris (Lille, France) and LC Laboratories (Woburn, MA, USA), respectively. MAY0132 and ESI-05 were synthesized by the laboratory of co-author J.Z. [43]. Poly I:C and recombinant human TNF-α were purchased from Sigma-Aldrich and R and D Systems (Minneapolis, MN, USA), respectively. 

### 2.2. Cytokine and Chemokine Quantification

A549 and SAE cells were inoculated with HMPV, AdV, or RSV at a multiplicity of infection (MOI) of 1. After 2 h, cells were treated with dimethyl sulfoxide (DMSO, vehicle control), 20 μM CE3F4, or 20 μM MAY0132 in fresh media. At 15 h post-treatment, cytokines/chemokines from collected supernatant were quantified using a Bio-Plex Pro Human Cytokine 27-plex Assay kit from Bio-Rad according to the manufacturer’s instructions. Data were analyzed using the multiplex analysis software from Bio-Rad.

### 2.3. Quantitative Real-Time PCR (qRT-PCR)

For quantitation of cytokine/chemokine mRNAs or the mRNA of AdV-DNA-binding protein (DBP), total cellular RNA was extracted using TRIzol reagents (Thermo Fisher Scientific, Waltham, MA, USA), followed by reverse transcription into cDNA using iScript^TM^ cDNA Synthesis Kit (Bio-Rad, Hercules, CA, USA) according to the manufacturer’s instructions. qRT-PCR was performed by using iTaq™ Universal SYBR Green Supermix (Bio-Rad, Hercules, CA, USA) in the CFX Connect Real-Time PCR System (Bio-Rad, Hercules, CA, USA). To quantify the viral genome or N gene of HMPV or RSV, total cellular RNA was extracted and cDNA was synthesized using HMPV- or RSV-specific reverse transcription primers with TaqMan™ Reverse Transcription Reagents from Thermo Fisher Scientific, followed by qRT-PCR, as described in [44,45]. The expression of IP-10, RANTES, AdV-DBP, and HMPV- or RSV-N was calculated by the 2^−^^ΔΔct^ method. 18S ribosomal RNA or glyceraldehyde 3-phosphate dehydrogenase (GAPDH) was used for normalization. The amount of HMPV or RSV genome was calculated by the absolute quantitation method. The information on primers for reverse transcription and qRT-PCR is shown in Supplementary Table S1.

### 2.4. Reporter Gene Assay

293 cells in triplicate were transfected with a luciferase reporter gene plasmid containing multiple copies of NF-κB binding sites (NF-κB-luc) using X-tremeGENE 9 (Roche, Indianapolis, IN, USA), as described previously [33,40], treated with or without the EPAC-isoform-specific inhibitors. Dimethyl sulfoxide (DMSO) was used as a vehicle control. At 15 h post-transfection, cells were transfected with poly I:C using Lipofectamine 2000 (Life Technologies, Grand Island, NY, USA) or treated with TNF-α with or without the inhibitor. After 6 h, cells were lysed to measure luciferase reporter activity. Cells without poly I:C or TNF-α treatment were used as negative controls. 

### 2.5. Statistical Analysis

Statistical significance was determined using analysis of variance (ANOVA). A *p* value less than 0.05 was considered significant. One and two asterisks designate a *p* value less than 0.05 and 0.01, respectively. Data are presented as means ± standard error (SE).

## 3. Results

### 3.1. EPAC2 Promotes HMPV Replication

Our early observation on the EPAC2′s significance in RSV infection prompted us to investigate the broad effect of EPAC on other respiratory viral infections [33]. To the best of our knowledge, the function of EPAC2 in viral infection has not been extensively demonstrated. EPAC1, on the other hand, has been shown to be involved in MERS-CoV, Ebola virus, and recently vesicular stomatitis virus (VSV) infections [28,34,35]. However, the overall impact of EPAC1 on viral infections, especially the regulatory mechanisms, is still largely unknown. 

**Figure 1:**
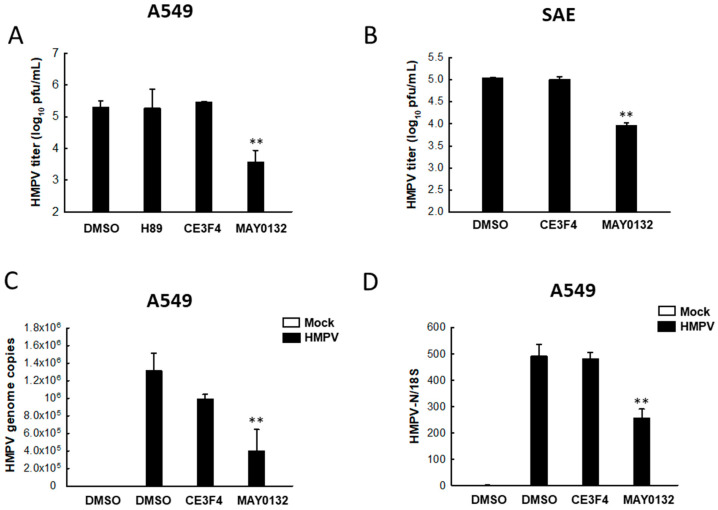
The impact of EPAC2 on HMPV replication. (**A**,**B**) A549 (**A**) or SAE (**B**) cells in triplicate were infected with HMPV at an MOI of 1. At 2 h post-infection (p.i.), cells were washed and subsequently treated with 20 μM CE3F4 or MAY0132. Next, 20 μM H89 was used to examine the role of cAMP/PKA signaling in HMPV infection. DMSO was used as vehicle control for EPAC inhibitors. At 15 h post-treatment, total viruses were harvested, and viral titer was determined by immunostaining using an anti-HMPV antibody. Data are representative of three independent experiments. ** *p* < 0.01, relative to the DMSO-treated group. (**C**,**D**) A549 cells in triplicate were infected with HMPV, followed by drug treatment as described in (**A**). After 15 h post-treatment, total RNA was extracted and subjected to qRT-PCR to measure HMPV genome copies (**C**) and N gene expression (**D**). 18S ribosomal RNA (rRNA) was used for normalization to N gene level. DMSO-treated mock infection was used as a negative control for DMSO-treated HMPV infection. ** *p* < 0.01, relative to the DMSO-treated and HMPV-infected group.

Although HMPV and RSV belong to the same family (Pneumoviridae) and share many things in common, they also carry their distinct features [46,47]. To investigate whether and how EPAC plays a critical role in HMPV infection, we decided to investigate the impact of CE3F4 and MAY0132 on HMPV replication. We also tested a PKA inhibitor (H89) to explore whether cAMP/PKA signaling is involved in viral replication as well. As shown in Figure 1A, the MAY0132 treatment of A549 cells, a human alveolar epithelial cell line, led to a significant decrease in HMPV titer, by one and a half log, compared with DMSO-treated cells. Either H89 or CE3F4 treatment seemed uninfluential on HMPV replication. We also observed the inhibitory effect of MAY0132 on HMPV replication in primary small airway epithelial (SAE) cells, a more physiologically relevant cell model for the infection. Similar to the result demonstrated in Figure 1A, MAY0132-treated SAE cells showed remarkable suppression of infectious virus particles, compared with DMSO treatment, while CE3F4 treatment had no effect (Figure 1B). Furthermore, we confirmed the anti-HMPV effect of MAY0132 by investigating HMPV genome copies and N gene expression using qRT-PCR. Treatment with MAY0132, but not CE3F4, resulted in a considerable suppression in viral genome copies (Figure 1C) and N expression (Figure 1D) in comparison with the infected samples with DMSO treatment. Overall, these results demonstrate that EPAC2 is the isoform that is involved in the regulation of HMPV replication in airway epithelial cells (ACEs), a cell line, or primarily cultured cells.

### 3.2. EPAC2 Regulates HMPV-Induced Cytokines/Chemokines

**Figure 2:**
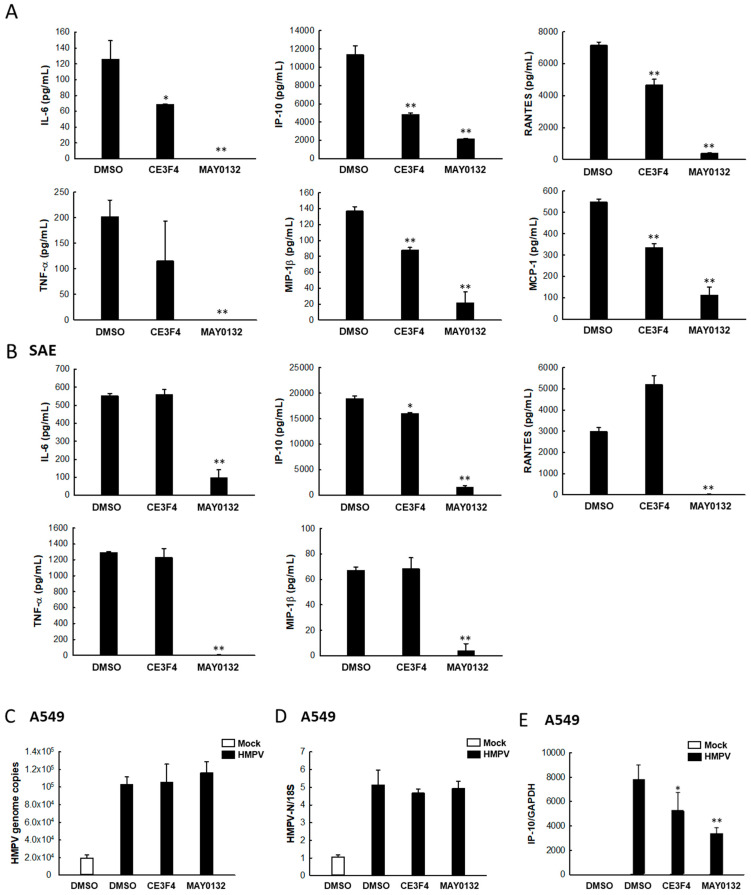
EPAC2-mediated cytokine/chemokine induction by HMPV. (**A**,**B**) A549 (**A**) or SAE (**B**) cells in triplicate were infected with HMPV at an MOI of 1. At 2 h p.i., cells were treated with DMSO, 20 μM CE3F4, or 20 μM MAY0132. At 15 h post-treatment, the supernatant was collected, and the amount of diluted or undiluted cytokines/chemokines was measured by Bio-Plex. * *p* < 0.05 and ** *p* < 0.01, relative to the DMSO-treated group. (**C**–**E**) A549 cells in triplicate were infected with HMPV, followed by the drug treatment as described in (**A**). At 4 h post-treatment, total RNA was extracted and subjected to qRT-PCR to measure HMPV genome copies (**C**), N gene expression (**D**), and IP-10 production (**E**). DMSO-treated mock infection was used as a negative control for DMSO-treated HMPV infection. * *p* < 0.05 and ** *p* < 0.01, relative to the DMSO-treated and HMPV-infected group.

The innate immune response is the first line of defense of hosts against viral infection and cytokines/chemokines are key molecules in innate immunity. HMPV has been demonstrated as a strong inducer of cytokines/chemokines [47,48] and several pro-inflammatory mediators are associated with increased disease severity in HMPV-infected patients [49,50]. To examine whether EPAC2 regulates virus-induced cytokines/chemokines, we also compared HMPV-induced cytokines/chemokines among cells, treated with/without chemical compounds. We found that, in A549 cells, HMPV-induced pro-inflammatory immune mediators such as IP-10, RANTES, MCP-1, TNF-α, MIP-1β, and IL-6, were significantly decreased by MAY0132 treatment, compared with that of DMSO (Figure 2A). Figure 2A also showed that CE3F4 suppressed the production of these cytokines/chemokines in response to HMPV infection but to a lesser extent than that of MAY0132. Consistent with the anti-inflammatory impact of MAY0132 on HMPV-infected A549, MAY0132-treated SAE cells also showed significantly suppressed production of IL-6, IP-10, RANTES, TNF-α, and MIP-1β, compared with DMSO control cells (Figure 2B). However, CE3F4 did not suppress the immune mediators’ induction in SAE cells, demonstrating a slight difference in cellular responses between an infected airway epithelial cell line and primary cultured airway epithelial cells and also highlighting the importance of EPAC2 in HMPV infection.

In the context of viral infection, the production of cytokines/chemokines is often dependent on viral replication [46,51], but sometimes the induction could also be replication independent [52,53]. To determine whether MAY0132-decreased cytokine/chemokine induction completely resulted from decreased viral replication, we measured cytokines/chemokines at 4 h post-infection (p.i.), an early time point when the viral replication (Figure 2C) and the N expression (Figure 2D) in the DMSO-, CE3F4-, and MAY0132-treated cells were comparable. At the early time point post-infection, most HMPV-induced cytokines/chemokines are too little to be detected by real-time PCR. IP-10, however, is a very inducible molecule and is the most inducible protein at 15 h p.i. Therefore, we used the IP-10 expression as a representative to study the impact of EPAC on HMPV-induced inflammatory gene expression. As shown in Figure 2E, at the early time point p.i., HMPV-induced IP-10 was detectable and significantly suppressed by MAY0132 treatment. CE3F4 also decreased virus-induced IP-10 transcription but less effectively than MAY0132. IP-10 is a suitable representative of important immune mediators for the study, as it is associated with the severity of acute respiratory infection in healthy adults [54]. In addition, IP-10 production has also been shown in rhinovirus-infected monocytic and reovirus-infected epithelial cells [55,56]. Overall, our results suggested that EPAC2 could control early inflammatory responses to HMPV infection in a replication-independent fashion. 

### 3.3. EPAC2 Is Responsible for AdV Replication

**Figure 3:**
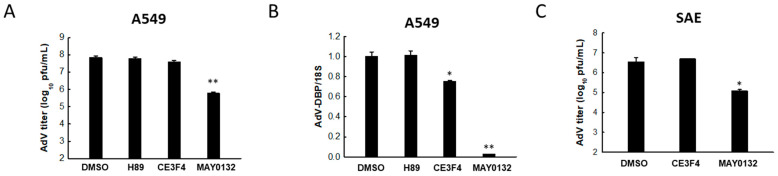
The impact of EPAC2 on AdV replication. (**A**) A549 cells in triplicate were infected with AdV at an MOI of 1. At 2 h p.i., cells were washed, followed by treatment with DMSO, 10 μM H89, 10 μM CE3F4, or 10 μM MAY0132. At 48 h post-treatment, total viruses were harvested, and infectious AdV particles were quantified by the plaque assay using crystal violet staining. Data are representative of three independent experiments. ** *p* < 0.01, relative to the DMSO-treated group. (**B**) A549 cells in triplicate were infected with AdV and treated with drugs as described in (**A**). At 48 h post-treatment, total RNA was extracted and subjected to qRT-PCR to measure AdV-DBP gene expression. 18S rRNA was used for normalization. * *p* < 0.05 and ** *p* < 0.01, relative to the DMSO-treated group. (**C**) SAE cells were infected with AdV at an MOI of 1 and then treated with DMSO, 10 μM CE3F4, or 10 μM MAY0132. At 48 h post-treatment, total viruses were prepared, and infectious AdV particles were quantified by the plaque assay. Data are representative of three independent experiments. *p < 0.05 and **p < 0.01, relative to the DMSO-treated group.

As RSV, HMPV, Ebola, VSV, and MERS-CoV are all RNA viruses, we then decided to take a look at whether EPAC plays a role in respiratory DNA viral infection. Herein, AdV, which is a double-stranded DNA virus and also one of the predominant viral pathogens causing lower RTI in humans, was used for the study [16]. As shown in Figure 3A, the AdV titer was not affected by treatment with H89 and CE3F4, while there was a significant drop in viral replication in the MAY0132-treated A549 cells, compared with DMSO-treated infected cells. Furthermore, MAY0132 showed an inhibitory effect on the expression of the AdV-DBP gene, which is essential for AdV DNA replication and gene expression [57] (Figure 3B). CE3F4 slightly decreased AdV-DBP gene expression, although it did not affect AdV titer. The anti-AdV function of MAY0132 was also confirmed in virus-infected SAE cells, which shows the suppression in infectious progeny virus (Figure 3C). These results demonstrate that EPAC2 inhibition also suppresses the replication of AdV, which is a respiratory DNA virus.

### 3.4. EPAC2 Regulates AdV-Induced Cytokines/Chemokines

**Figure 4:**
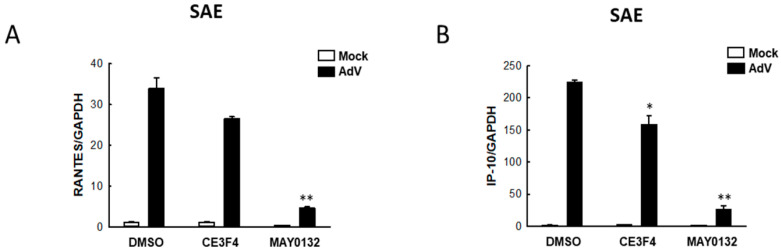
EPAC2-mediated chemokine induction by AdV. (**A**,**B**) SAE cells in triplicate were mock-infected or infected with AdV at an MOI of 1. At 2 h p.i., cells were treated with DMSO, 10 μM CE3F4, or 10 μM MAY0132. At 48 h post-treatment, total RNA was extracted and subjected to qRT-PCR to measure the expression of RANTES (**A**) and IP-10 (**B**). GAPDH was used for normalization. * *p* < 0.05 and ** *p* < 0.01, relative to the DMSO-treated and AdV-infected group.

Next, we examined whether MAY0132 affects cytokine/chemokine production upon AdV infection by measuring the expression of RANTES and IP-10, two pro-inflammatory chemokines accounting for the severity of acute respiratory infection [54,58]. Compared with RSV and HMPV, AdV is not a great inducer of cytokines/chemokines. Our AdV belongs to type 5, which has been reported as a weak inducer of cytokines/chemokines compared to other AdV serotypes [59,60], but can induce RANTES and IP-10 in human respiratory cells [61]. As shown in Figure 4, AdV infection of SAE cells led to the induction of RANTES and IP-10. MAY0132 significantly blocked both AdV-induced pro-inflammatory mediators, whereas CE3F4 also had a minor impact on IP-10 induction. Overall, these results demonstrate that EPAC2 inhibition has a significant anti-inflammatory effect on AdV infection. 

### 3.5. EPAC2 Regulates RSV Replication and Virus-Induced Cytokines/Chemokines

**Figure 5:**
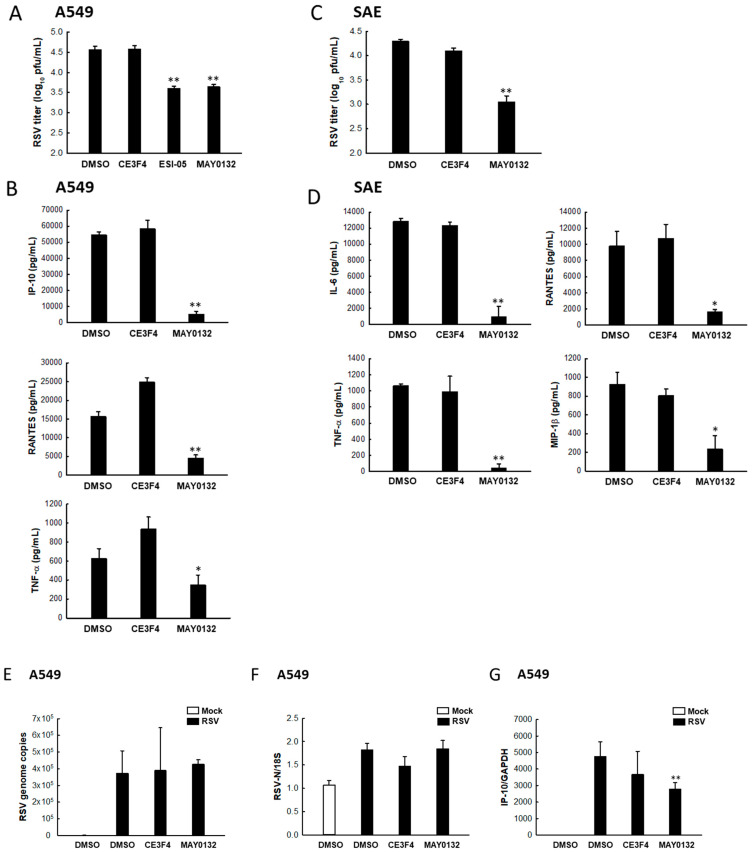
The impact of EPAC2 on RSV infection. (**A**,**B**) A549 cells in triplicate were infected with RSV at an MOI of 1. After 2 h p.i., cells were treated with DMSO, 20 μM CE3F4, 20 μM ESI-05, or 20 μM MAY0132. At 15 h post-treatment, (**A**) total viruses were collected, and viral titer was determined by immunostaining using an anti-RSV antibody. (**B**) The supernatant was collected, and diluted or undiluted cytokines/chemokines were measured by Bio-Plex. * *p* < 0.05 and ** *p* < 0.01, relative to the DMSO-treated group. (**C**,**D**) SAE cells were infected with RSV and treated with the indicated drugs as described in (**A**). At 15 h post-treatment, (**C**) virus titration was determined. ** *p* < 0.01, relative to the DMSO-treated group. (**D**) Cytokines/chemokines of SAE cells were also measured by Bio-Plex. * *p* < 0.05 and ** *p* < 0.01, relative to the DMSO-treated group. (**E**–**G**) A549 cells in triplicate were infected with RSV and treated with drugs as described in (**A**). At 4 h post-treatment, total RNA was extracted and subjected to qRT-PCR to measure RSV genome copies (**E**), N gene expression (**F**), and IP-10 transcription (**G**). DMSO-treated mock infection was used as a negative control for DMSO-treated RSV infection. * *p* < 0.05 and ** *p* < 0.01, relative to the DMSO-treated and RSV-infected group.

We have previously revealed that treatment with ESI-05, another EPAC2-specific inhibitor, suppresses RSV replication and pro-inflammatory responses [33]. Consistently, we found that treatment with MAY0132 led to the remarkable suppression of RSV replication in A549 cells, which is comparable to that of ESI-05 (Figure 5A). Herein, we also found that CE3F4 treatment did not show an antiviral effect, consistent with our publication on an unessential role of EPAC1 in RSV replication based on EPAC1 knockdown experiments [33]. As shown in Figure 5B, MAY0132 also reduced the production of virus-induced immune mediators such as IP-10, RANTES, and TNF-α [33]. EPAC2-mediated RSV replication and cytokine/chemokine induction by RSV were also confirmed in SAE cells (Figure 5C,D). We also found that IP-10 induction was impaired by MAY0132 treatment early after RSV infection (Figure 5G) when the change of viral genome (Figure 5E) and N gene (Figure 5F) by inhibitors could not be detected, suggesting replication-independent direct suppression of MAY0132 on cytokine induction. Taken together, these results support our previous findings on the importance of EPAC2, but not EPAC1, in regulating RSV infection. 

### 3.6. EPAC2 Regulates Poly I:C- and TNF-α-Induced Inflammatory Signaling Pathways

**Figure 6:**
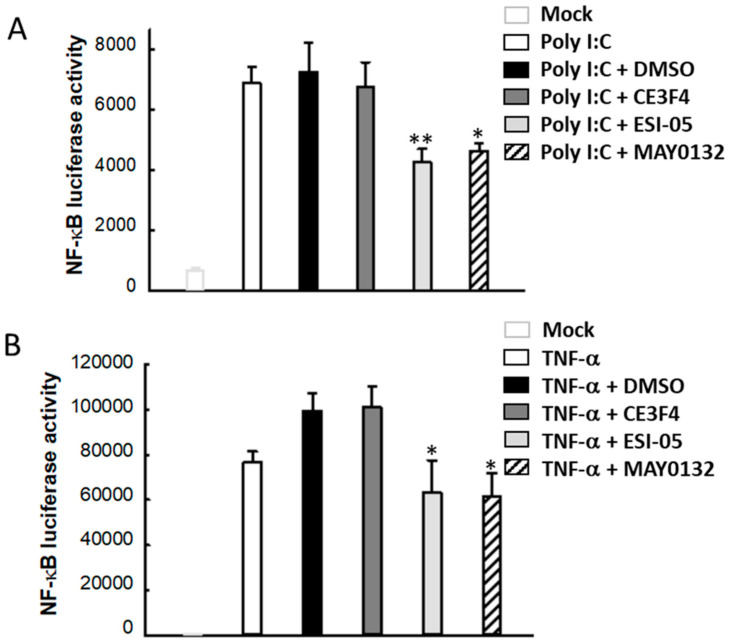
The EPAC2-mediated NF-κB activation by poly I:C or TNF-α. (**A**,**B**) 293 cells in triplicate were transiently transfected with a luciferase reporter plasmid containing the NF-κB promoter (NF-κB-luc) in the presence of the indicated drugs. At 15 h post-transfection, cells were transfected with 1 μg/mL poly I:C (**A**) or treated with 10 ng/mL of TNF-α (**B**) for an additional 6 h. Cells were then lysed to measure luciferase activities. Cells without poly I:C or TNF-α treatment were used as a negative control. Data are representative of three independent experiments. * *p* < 0.05 and ** *p* < 0.01, relative to the poly I:C-transfected and DMSO-treated group or the TNF-α- and DMSO-treated group, respectively.

NF-κB is one of the major transcription factors that control the expression of cytokines/chemokines [62,63]. In response to viral infections, the induction of various inflammatory genes requires NF-κB activation for their transcription [64,65]. Since EPAC2 seems to play a dominant role in HMPV, AdV, and RSV infection, and the EPAC2-mediated cytokine/chemokine induction likely has a component that is replication independent, we used MAY0132 to investigate the role of EPAC2 in poly I:C/ TNF-α-activated NF-κB. As shown in Figure 6A,B, when cells were transfected with a luciferase reporter plasmid containing the binding sites of NF-κB, treatment with poly I:C or TNF-α efficiently increased the luciferase activity, indicating NF-κB activation. The addition of ESI-05 or MAY0132 significantly decreased poly I:C- and TNF-α-enhanced signals, supporting that EPAC2 mediated poly I:C/ TNF-α-activated inflammatory signaling. 

## 4. Discussion

Respiratory viral-induced illnesses are among the most common and severe diseases in the pediatric age group and high-risk populations, resulting in a great need for effective treatment and preventive vaccines. This study aims to evaluate the function of EPAC in both RNA and DNA respiratory viral infections using isoform-specific inhibitors. Herein, we demonstrated that EPAC2 plays a dominant role in regulating HMPV, AdV, and RSV infections. Treatment with MAY0132, an EPAC2-specific inhibitor, suppressed not only viral replication but also the induction of pro-inflammatory mediators in response to these three viruses. Moreover, the anti-inflammatory effect of MAY0132 was also observed in the context of poly I:C and TNF-α stimulation. Taken together, this study provides the first evidence on the potential of EPAC2 as a broad-spectrum drug target of multiple respiratory viral infections. 

As discussed, EPAC has two major forms: EPAC1 and EPAC2. They exhibit distinct expression patterns and interacting partners, suggesting the discrete involvement in diverse biological functions [66,67]. The development of EPAC-isoform-specific pharmacological modulators led to significant progress in studying EPAC-mediated signaling pathways [30,31,32]. CE3F4 and its derivative have been well known as EPAC1 inhibitors [68], and AM-001 was recently identified an EPAC1 inhibitor as well [69]. In addition, EPAC2-specific inhibitors including ESI-05, HJC0350, and MAY0132 have been also developed [67]. Among them, MAY0132 has been well-characterized as a specific and potent EPAC2 inhibitor with an apparent 50% inhibitory concentration (IC_50_) value of 0.4 μM [43]. It selectively inhibits EPAC2 with no effect on EPAC1 activity at up to 100 μM. Herein, we used EPAC inhibitors to our benefit to discover EPAC2 as a new determinant to control the viral replication and host innate responses to HMPV and AdV. In this study, we also found that the EPAC1 inhibitor CE3F4 also suppressed some immune mediators in HMPV and AdV infections, however, to a lesser extent and significance than the EPAC2 inhibitors. Using EPAC1/2-specific inhibitors we also confirmed the role of EPAC2 and excluded the involvement of EPAC1 in RSV infection [33]. Therefore, these studies demonstrated that EPAC2 has an overall prevalent function in HMPV, AdV, and RSV infections. As discussed, some viruses, including MERS-CoV and Ebola, use EPAC1 to promote entry [28,35], supporting that EPAC isoforms and associated signaling pathways are also viral specific. How viruses select EPAC isoforms to evade host defense needs to be further elucidated in the future. 

The development of diagnostic methods such as next-generation sequencing and multiplex PCR has allowed for the simultaneous identification of multiple causative agents from one sample in rapid and accurate ways. Based on this technology, there have been accumulating observations that respiratory diseases are attributed to multiple respiratory pathogens and a significant portion of cases shows co-infections with more than one virus [70,71]. Currently, it is controversial whether viral co-infections are associated with the severity of clinical outcomes. Several groups have reported that co-infection is less severe than a single infection and even beneficial, possibly due to viral competition for resources [72,73,74,75]. On the contrary, other studies have reported the association between co-infection and severe disease outcomes including pediatric intensive care unit (PICU) admission [15,76,77,78]. Despite the controversy, it is evident that drugs targeting multipathogens during viral co-infections should be beneficial for respiratory illness control. HMPV, AdV, and RSV are among the most common contributors to prevailing human respiratory disease, with co-infections that occur quite often as well [79,80]. Since EPAC2-specific inhibitors have a broad antiviral and anti-inflammation impact against all three major respiratory viruses, it is a promising treatment of modality against HMPV, AdV, and RSV.

As mentioned above, the activation of NF-κB is essential for the induction of inflammatory and antiviral genes [62]. The NF-κB pathway is activated in response to diverse stimuli including pattern-recognition receptors (PRRs) such as Toll-like receptors (TLRs) and retinoic acid-inducible gene I (RIG-I)/MDA5, as well as, TNF receptor family members [81]. Previous findings have revealed that HMPV and RSV infections of AECs induce the first wave of immune mediators through the NF-κB signaling pathway, which is activated via RIG-I/mitochondrial antiviral signaling protein (MAVS)-dependent signaling pathways [81,82]. On the other hand, poly I:C, a well-characterized inducer of NF-κB, can be recognized through two different pathways of TLR-3 and RIG-I/MDA5 [83,84,85]. In this study, since 293 cells do not express any TLRs, NF-κB activation by poly I:C treatment of 293 cells (Figure 6) is possibly from RIG-I/MDA5 or other unrevealed signaling [86]. Given the fact that MAY0132 efficiently blocked NF-κB activation, EPAC2-mediated signaling might either interact with the downstream molecules along with NF-κB-activating pathways or directly target NF-κB. In the future, we will investigate the molecules associated with EPAC2 and understand the consequence of such an interaction in regulating viral replication and host innate immune responses to respiratory viral infections. We are also planning to expand our research to different virus serotypes to investigate whether the impact of EPAC on the virus is serotype specific.

## Data Availability

All data is available upon the request.

## Supplementary Materials

The following are available online at https://www.mdpi.com/article/10.3390/v13061179/s1. Table S1. Primers for reverse transcription and qRT-PCR.
