# Supplementary material for: Broad Impact of Exchange Protein Directly Activated by cAMP 2 (EPAC2) on Respiratory Viral Infections
Source: Viruses. 2021 Jun 21;13(6):1179. doi: 10.3390/v13061179 (PMC8233786; doi:10.3390/v13061179)
Supplement: Supplementary file 1 [file viruses-13-01179-s001.zip › viruses-1219849-supplementary.pdf]

viruses

Supplementary Materials: Broad Impact of Exchange Protein Directly Activated by cAMP 2 (EPAC2) on Respiratory Viral Infections

Table S1. Primers for reverse transcription and qRT-PCR.

Primers for Reverse Transcription		
**Name**		**Sequence (5' to 3')**
HMPV genome		CGTCTCAGCCAATCCCTGGTGATTATGAGTAATTAAAAAAVTGGGACAAG
RSV genome		CTGCGATGAGTGGCAGGCACTACAGTGTATTAGACTTRACAGCAGAAG
HMPV-N		CGTCTCAGCCAATCCCTGGTTTTTTTTTTTTTAATTACTC
RSV-N		CTGCGATGAGTGGCAGGCTTTTTTTTTTTTTAACTYAAAGCTC
**Primers for qRT-PCR**		
**Name**		**Sequence (5' to 3')**
HMPV genome	Forward	GCTTCATTACCCATGAAMAGAATATC
	Reverse	CGTCTCAGCCAATCCCTGG
RSV genome	Forward	GCATCTTCTCCATGRAATTCAGG
	Reverse	CTGCGATGAGTGGCAGGC
HMPV-N	Forward	CACAGARCTATTTTCWGCAGCAG
	Reverse	CGTCTCAGCCAATCCCTGG
RSV-N	Forward	ACTACAGTGTATTAGACTTRACAGCAGAAG
	Reverse	CTGCGATGAGTGGCAGGC
AdV-DBP	Forward	GCCATTGCGCCCAAGAAGAA
	Reverse	CTGTCCACGATTACCTCTGGTGAT
IP-10	Forward	GTGGCATTCAAGGAGTACCTC
	Reverse	GCCTTCGATTCTGGATTCAGACA
RANTES	Forward	CCTGCTGCTTTGCCTACATTGC
	Reverse	ACACACTTGGCGGTTCTTTCGG
18S	Forward	ACATCCAAGGAAGGCAGCAG
	Reverse	TCGTCACTACCTCCCCGG
GAPDH	Forward	CTCAAGATCATCAGCAATGCCT
	Reverse	AAGTTGTCATGGATGACCTTGG
